# The impact of the work environment on the health-related quality of life of Licensed Practical Nurses: a cross-sectional survey in four work environments

**DOI:** 10.1186/s12955-022-01951-9

**Published:** 2022-03-19

**Authors:** Leah Adeline Phillips, Nyla de Los Santos, Henry Ntanda, Jennifer Jackson

**Affiliations:** 1 Alberta College of Family Physicians, #370, 10403-172 Street, Centre 170, Edmonton, AB T5S 1K9 Canada; 2 College of Licenced Practical Nurses of Alberta, St. Albert Trail Place, 12163-146 Street, Edmonton, AB Canada; 3 Cumming School of Medicine, Department of Paediatrics, University of Calgary, Calgary, AB T2N 1N4 Canada; 4 Faculty of Nursing, University of Calgary, Professional Faculties Building, 2500 University Drive NW, Calgary, AB T2N 1N4 Canada

**Keywords:** Nurses, Work environment, Health-related quality of life, Resilience, Survey

## Abstract

**Background:**

Nurses are known to have negative health outcomes related to their work. While it is acknowledged that nursing work is associated with things like back injuries and burnout, there is limited evidence as to what factors in the work environment contribute to these issues.

**Purpose:**

The aims of this study were to assess how Licensed Practical Nurses (LPNs) report their Health-related quality of life (HRQoL), and how nurses’ health is impacted by their work environment.

**Methods:**

These data used for analysis comes from a cross-sectional survey administered online to all LPNs in Alberta (2018). The survey collected data on the following variables: participant’s demographics, the SF-36 HRQoL, Practice Environment Scale of the Nursing Work Index (PES-NW) and the CD-RISC measure of resilience. The beta distribution was used to model HRQoL outcomes. In instances where optimal health (score of ‘1’) was observed then an extended version of beta distribution (called one—inflated beta) was applied.

**Results:**

4,425 LPNs responded to the survey. LPNs (mean age: 40) report lower scores on each SF-36 subscale than the general Canadian population aged 35–44. LPNs who work ‘causal’ had better physical health, (OR 1.21, CI 1.11–1.32, p = 0.000), and mental health (OR 1.22, CI 1.12–1.30, p = 0.000) than LPNs who work full time, even after controlling for resilience. LPNs’ views on the adequacy of staffing and resources in their workplaces have an influence across all dimensions of health.

**Conclusion:**

This study suggests that improvements in the work environment could positively impact health outcomes and that adequate resourcing could support the nursing workforce.

**Supplementary Information:**

The online version contains supplementary material available at 10.1186/s12955-022-01951-9.

## Introduction

Nurses are essential for safe healthcare delivery, including patient care. In order to support the health of others, nurses need to be well themselves. It is well established that improvements in healthcare workplaces, such as increased staffing, improve patient outcomes [2, 3]. However, assessments of nurses’ health have frequently focused on individual level factors, including areas like diet and exercise [18, 22, 39]. Fewer studies have examined how nurses’ work environments impact upon their health. This study examined the impact of work environment on nurses’ Health Related Quality of Life (HRQOL).

## Background

Work environment is known to influence workers’ health in many sectors, and nurses are no exception. Research shows that nurses are at increased risk for musculoskeletal injuries, particularly back injuries [15, 16, 19, 38]. Additionally, nurses have higher injury rates than workers in agriculture, mining, or construction [47]. Problems arising from shift work, such as sleep disturbances, fatigue, and depression can increase nurses’ intentions to leave their jobs [26]. The long hours and physical demands of nursing can cause irregular and abnormal menstruation [29] and difficulty becoming pregnant [20]. A 2015 nurses’ health study found that nurses experience many negative health consequences [8, 12, 20, 34, 45]. While it is evident that nursing is a physically taxing profession, there have been fewer studies exploring the impact of the work environment characteristics on nurses, rather than the work itself. This study assessed the self-reported health of Licensed Practical Nurses (LPN) and the impact of their work environments on their health. Licensed Practical Nurses are a regulated nursing profession in Canada and work in collaboration with members of health care teams [10].

## Research questions

The questions addressed in this study were:

1. How do LPNs rate their Health Related Quality of Life?
2. What is the impact of work environment on self-reported Health Related Quality of Life for LPNs, when controlling for resilience and demographic factors?

## Methodology

A cross-sectional survey of the population of LPNs in the Canadian province of Alberta was conducted in August 2018. The online survey was designed to take a well-rounded look at the work-life of Alberta LPNs. This article presents the study of the relationship between work environment, measured by the Practice Environment Scale of the Nursing Work Index (PES-NWI) (participation, leadership, resources, and relationships), personal resilience, and health-related quality of life, as measured by the Short Form 36 item Health Survey (SF-36).

### Procedure

The survey was distributed by email to the total population of Alberta’s LPNs (n = 15,860). LPNs were informed that their participation was voluntary, anonymous, and that completion of the survey implied consent. The survey was conducted by the Alberta LPN regulatory college and university partners. These data for this article were a secondary use of this original data. After discussion with the Community Ethics Research Board, it was determined that this secondary data analysis study was a low ethical risk and did not require a formal ethics review.

### Measures

#### Health-related quality of life

The SF-36 [48] examines general health, including both physical and emotional well-being. The SF-36 examines 8 dimensions of health, with each dimension is scored from 0–100. In general, higher scores indicate better health and functioning. The 8 dimensions can be collapsed into two summary scores referred to as the Physical Component Summary (PCS) and the Mental Component Summary (MCS). In studies of the general population, internal consistency reliability coefficients ranged from 0.92 to 0.94 for the PCS score and 0.87 to 0.89 for the MCS score [48].

#### Perceptions of work environment

Nursing practice environment is defined as the “organizational characteristics of a work setting that facilitate or constrain professional nursing practice” [27, p. 178]. The PES-NWI examines nurses’ perceptions of the various practice environments at their current job. The ‘Nurse Participation’ subscale refers to perceived opportunities for nurses to meaningfully participate in workplace activities, including at the broader organizational context (e.g., participate in policy decisions, serve on organizational committees, etc.). ‘Leadership’ refers to nurses’ perceptions of effective management and how well they are supported by management. The ‘Resources’ subscale measures their perception on staffing and resources available to do their work. The ‘Relationships’ subscale measures their perceptions of the quality of their professional relationships. The ‘Nursing Foundations’ quality of care’ subscale was not used in the present study. Lake [27] notes that the ‘Nursing Foundations’ subscale measures facility-level phenomena while the other subscales measure unit or ward-level phenomena. The four PES-NWI sub-scales used in this study assess how nurses feel about their immediate work environment.

The PES-NWI is a 4-point Likert scale. Respondents are asked to indicate the degree to which they feel each item is present in their current work environment. Items are scored from 1 (*strongly disagree*) to 4 (*strongly agree*). All the items in a subscale are summed and then the mean is calculated to produce a subscale score. A subscale score greater than 2.5 indicates a more positive perception of the work environment. The Cronbach’s alpha for the PES-NWI subscales have been reported as 0.89 for participation, 0.71 for leadership/manager, 0.77 for resources, and 0.85 for relationships [37].

#### Resilience

Resilience is defined as “the personal qualities that enable one to thrive in the face of adversity” [13], p. 76). The Connor-Davidson Resilience Scale (CD-RISC) is a widely used measures of resilience. The CD-RISC 10 is used in the present study. The CD-RISC 10 is a Likert scale with each item scored from 0 to 4. A respondent is asked to indicate how much they agree that each statement applied to them over the past month. Possible responses range from ‘not true at all’ (0) to ‘true nearly all the time’ (4). The scores for the 10 items are summed to produce an overall resilience score with the highest possible score of 40. Higher scores indicate greater resilience. The CD-RISC 10 is reported to have strong internal consistency with a Cronbach’s alpha of 0.87 in a study of hospital employees [28], and 0.81 in a study of student nurses [4].

### Data analysis

The SF-36 has 8 dimensions examining health where each dimension scored from 0 to 100. Therefore, for intuitive interpretations, scores for the sub-scales were first converted to proportions. This was to align our analysis with studies that have examined HRQoL together with corresponding sub scales, where outcomes are usually bounded between 0 and 1 [14]. Here, individuals with a score of zero would be perceived to have poor health, while those with a score of one would be assumed to be in optimal health. Scores between 0 and 1 have a natural beta distribution [14]. The outcome is linked to covariates in a regression model which has two structures: (i) the mean and (ii) dispersion. The mean structure models the average HRQoL, while the dispersion structure adjusts for non-constance of variability in the data. In our analysis, the beta distribution was used to model each of the following HRQoL outcomes mental composite score, body pain, emotional well-being, general health, and energy fatigue.

In instances where majority in a sample are of optimal health (score of ‘1’), then an extended version of beta distribution (called one—inflated beta) is deemed appropriate to describe these data [14]. One inflated regression partitions the population into two: those with optimal health and those with suboptimal health. By extension, this results into a three-part model: (i) a logistic regression comparing the outcomes of those with optimal health versus those with suboptimal health (HRQoL < 100%), (ii) beta regression model examining HRQoL outcome in those with suboptimal health (HRQoL < 100%), and lastly (iii) the dispersion part. The one—inflated beta distribution was used to model each of the following HRQoL outcomes: role physical, social functioning, physical functioning, role emotional and physical composite score. For both models, logit link function was preferred to use as this would allow for the interpretation of parameter estimates in terms of odds ratios [14]. To assess model, we compared observed and fitted values for each model (see Additional file 1).

In both beta and one inflated regression model, the mean structure of model was selected as follows: first, we explored if any non-linearity existed between the HRQoL subscale outcomes and each of the continuous covariates, i.e., leadership, resource, relationship, resilience, and participant age. This was mainly examining whether higher order polynomials would be suitable (see Additional file 1: figure X & Y). The following variables leadership, resource, relationship, resilience, gender, area of work (acute, continuing, community and primary care), years of practice (0–5,6–15 and 16 plus years), employment status (full time, part time and casual) and participant age were used as predictors to examine the association with each outcome variable.

Secondly, for each outcome, given the relationship visualized (see Additional file 1: figure X & Y) linear, quadratic, and cubic terms were explored. The models were compared using AICs and loglikelihood ratio test. If the difference in AIC values was less than 1%, we proceeded with linear relationship with the outcome. In this analysis there was no variable selection as all covariates were predetermined bested on literature review and expert opinion and were not removed from the model if not found to be significant.

All the analysis a performed using R package ‘gamlss’ [43] and betareg [14].

## Results

A total of 4,425 LPNs participated in the study. The average age of respondents was 39 years, most self-identified as female (92%) female, had full/part time employment (76%), worked primary in acute and continued care (63%) and most had work experience of 6 + years (51.5%). Demographic data are presented in Table 1.

**Table 1:** Socio-demographic characteristics and descriptive variables for the survey

Variables	Frequency (n = 4425)	Percentages
Sex		
Female	4067	91.9
Male	308	7.0
Prefer not to answer	50	1.1
Employment Status		
Full-time	1732	39.1
Part-time	1626	36.7
Casual	687	15.5
Unemployed	380	8.60
Primary work setting		
Acute care	1678	37.9
Continuing care	1092	24.7
Community	712	16.1
Primary care/education/research	943	21.3
Years of practice		
Less than 2 years	740	16.7
2–5 years	1243	28.1
6–10 years	958	21.6
11–15 years	522	11.8
16–20 years	281	6.4
21 years or more	519	11.7
Missing	162	3.7
	Mean (SD)	
Participant age (years)	39.1 (11.5)	
Nursing work environment		
PES—participation	2.71 (0.77)	
PES—leadership	2.87 (0.81)	
PES—resources	2.50 (0.93)	
PES—relationships	3.24 (0.72)	
Health quality of life (SF-36)		
Physical functioning (PF)	87.39 (19.20)	
Role physical (RP)	80.82 (31.90)	
Role emotional (RE)	78.41 (33.97)	
Vitality (VT)	57.24 (20.36)	
Mental health (MH)	75.21 (16.08)	
Social functioning (SF)	77.18 (23.41)	
Bodily pain (BP)	74.04 (21.64)	
General health (GH)	71.05 (18.61)	
Physical component summary	313.47 (69.10)	
Mental component summary	287.96 (78.17)	
Resilience	31.11 (5.52)	

*Practice environment scale (PES)

**Fig. 1:**
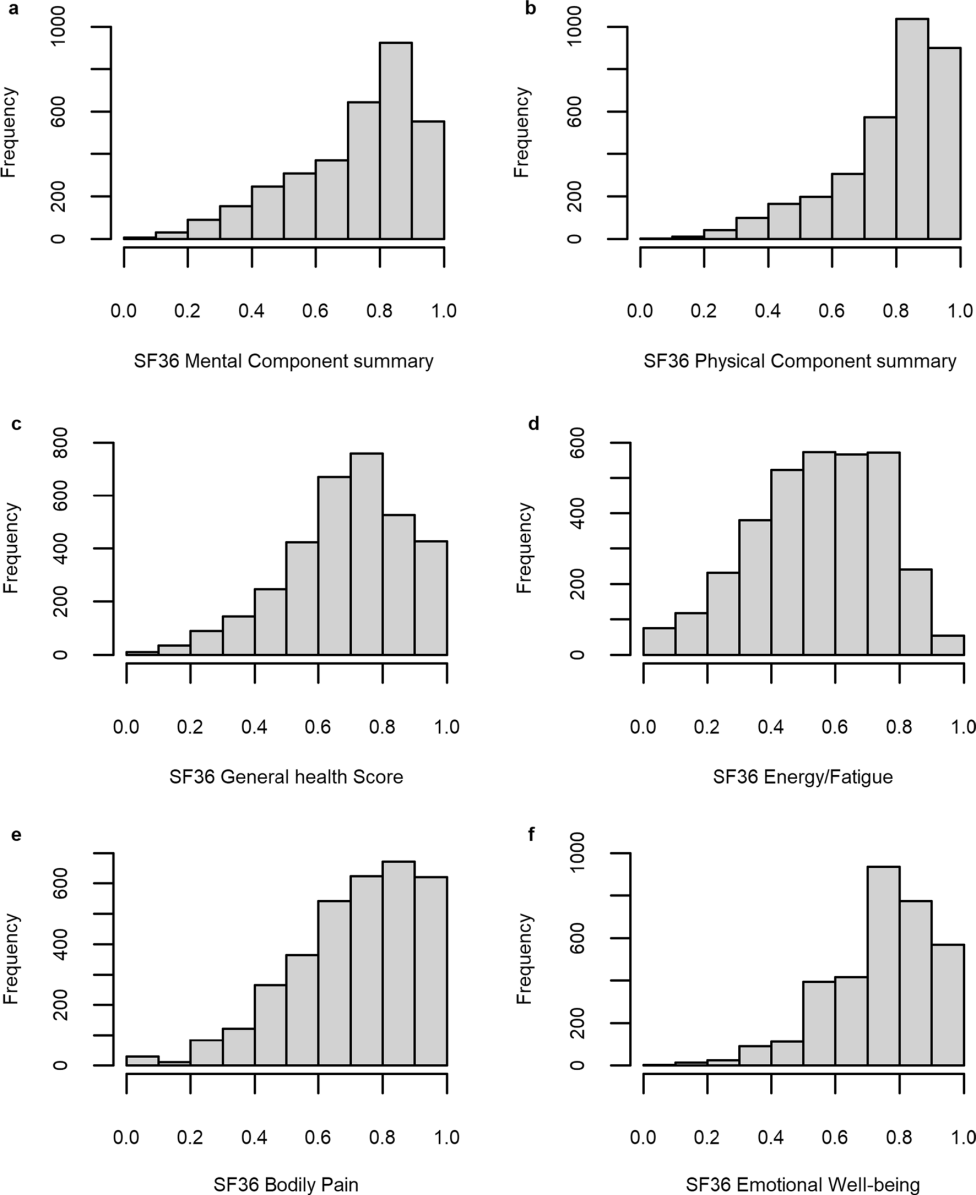

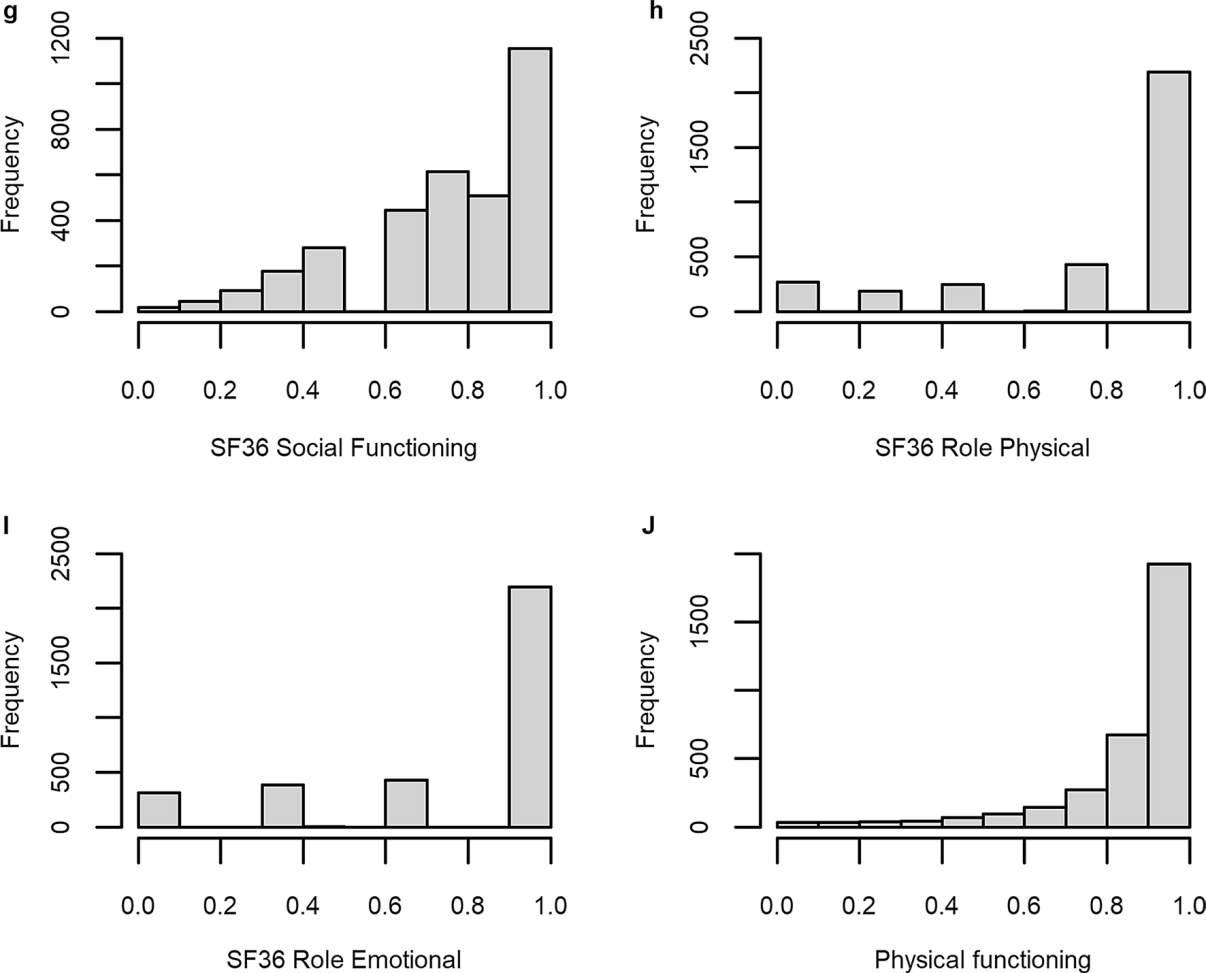
HRQoL response distribution

The responses to the SF-36 varied greatly across each subscale. Figure 1 illustrates the distribution of responses, with many subscales demonstrating a high degree of skewedness.

The regression estimates from both beta and one inflated regression are shown in Tables 2 and 3. In both models, resources, relationships, resilience, gender, and health status were all significantly associated with increased odds of mental component summary. Overall, resilience and resource are associated with higher odds of both physical and mental component summary for nurses. Employment status and relationships predicted increased mental functioning in nurses. Furthermore, resilience and participant gender were associated with increased physical component summaries for LPNs with optimal health.

**Table 2:** Predictors of mental component summary using a beta regression model

Covariates	Mean sub-model (logit link)**				Precision sub model (log link)		
	Odds ratio	95% CI	P-value	Estimate		Standard error	P-value
Intercept	0.08	0.06,0.10	**0.000**	1.180		0.183	**0.000**
Leadership	1.05	0.99,1.09	0.055	0.010		0.040	0.807
Resource	1.24	1.18,1.28	**0.000**	0.079		0.034	**0.021**
Relationship	1.05	0.99,1.10	0.071	0.053		0.042	0.210
Resilience	1.07	1.06,1.08	**0.000**	0.011		0.004	**0.011**
Male (ref: female)	1.08	0.96,1.21	0.190	0.002		0.095	0.980
Area of work (ref: acute care)							
Continuing care	1.02	0.95,1.09	0.548	0.022		0.062	0.720
Community care	0.93	0.85,1.01	0.081	− 0.008		0.070	0.905
Primary care	0.94	0.86,1.02	0.140	− 0.088		0.071	0.214
Year of practice (ref: 0–5 years)							
6–15 years	0.96	0.90,1.03	0.251	0.026		0.058	0.650
16 plus years	0.94	0.84,1.04	0.203	0.041		0.087	0.635
Employment status (ref: fulltime)							
Part time	1.09	1.02,1.15	**0.008**	0.032		0.053	0.538
Casual	1.22	1.12,1.30	**0.000**	0.163		0.069	**0.018**
Participant age	1.01	1.01,1.02	**0.000**	− 0.001		0.003	0.810

*Bolded,* Indicate statistical significance (at the 5% level)

*CI,* Confidence interval, Ref, Reference category

**Indicates predictors of suboptimal health using odds ratio

**Table 3:** Predictors of physical component summary using a one inflated beta regression model

Covariates	Mean (logit link)**		Sigma (log link)		NU (logit link) *	
	OR, 95% CI	P-value	Estimate (SE)	P value	Estimate (SE)	P-value
Intercept	0.49 (0.39,0.63)	0.000	1.473 (0.188)	0.000	− 10.4579 (1.3358)	0.000
Leadership	1.03 (0.98,1.08)	0.295	0.016 (0.041)	0.689	0.3509 (0.2625)	0.181
Resource	1.16 (1.11,1.21)	**0.000**	0.085 (0.035)	**0.017**	0.2132 (0.2036)	0.295
Relationship	1.08 (1.02,1.14)	**0.009**	0.082 (0.043)	0.061	0.5104 (0.3127)	0.103
Resilience	1.05 (1.04,1.05)	**0.000**	0.009 (0.004)	**0.045**	0.0998 (0.0291)	**0.001**
Male (ref: female)	1.05 (0.93,1.19)	0.456	0.010 (0.100)	0.924	0.8020 (0.3714)	**0.031**
Area of work (ref: acute care)						
Continuing care	1.02 (0.94,1.10)	0.627	0.088 (0.064)	0.169	− 0.2710 (0.3568)	0.448
Community care	1.03 (0.95,1.12)	0.452	0.133 (0.073)	0.070	− 0.3855 (0.4165)	0.355
Primary care	1.00 (0.90,1.09)	0.927	0.022 (0.075)	0.773	0.2865 (0.3598)	0.426
Year of practice (ref: 0–5 years)						
6–15 years	1.01 (0.93,1.09)	0.855	0.001 (0.062)	0.987	− 0.0452 (0.3166)	0.886
16 plus years	0.91 (0.81,1.02)	0.096	− 0.129 (0.092)	0.163	− 0.0765 (0.4848)	0.875
Employment status (ref: fulltime)						
Part time	1.07 (1.00,1.14)	0.052	0.007 (0.055)	0.903	− 0.2448 (0.3041)	0.421
Casual	1.21 (1.11,1.32)	**0.000**	0.021 (0.072)	0.770	− 0.0284 (0.3709)	0.939
Participant age	1.00 (0.99,1.00)	0.053	− 0.010 (0.003)	**0.000**	− 0.0031 (0.0152)	0.840

*Nu (logit link) indicates predictors of the optimal health in log-odds values

**Indicates predictors of suboptimal health using odds ratio

*SE *Standard errors, *OR *Odds ratios, *CI *Confidence interval

*Bolded,* Indicate statistical significance (at the 5% level);

Ref, Reference category

## Discussion

In this study, we assessed how LPNs rate their HRQoL, and what impact work environment had upon their HRQoL. We had a high proportion of female participants. This was expected, as nursing populations generally include > 90% female employees [49].

Overall, LPNs in this study (mean age: 40) rated their health as being lower than Canadian norms for people 35–44 [23] across every SF-36 subscale. However, this sample reported higher mean scores than some published examples of nurses’ SF-36 scores [6, 11, 42], and similar scores to those of Omrani and Talebi [36]. These results indicate that LPNs report their health as lower than the general population, but higher than other groups of nurses. Self-reported health is a key factor in healthcare worker retention, with eldercare employees with lower psychological wellbeing scores leaving their jobs more often than those with higher scores [21]. Nurses reporting increased health problems also reported increased rates of turnover [26].

LPNs reported that their workplace resources and employment status significantly impacted their HRQoL. It is well known that improvements in healthcare work environments improve patient outcomes [1, 9]. This study illustrates that improvements in the work environment can also impact nurse health outcomes. Quality work environments are associated with lower levels of nurse burnout [3]. The results of this study add that sufficient equipment, staffing, and other resources is positively related to all the dimensions of LPN health. This result could indicate that heaver work loads with fewer resources could be detrimental to the health of nurses, thereby putting patients at risk. Improvements in work environment will likely result in improved nurses’ health as well.

It is also important to note that work environments make a difference to nurses’ health, even after controlling for resilience. There have been a plethora of studies on nurses’ resilience [5, 7, 17, 24, 25, 30–33, 40, 41, 46]. These studies have been criticized for placing more emphasis on the individual, rather than considering nursing workforce issues [35, 44]. While personal resilience is a predictor of LPNs’ HRQoL, resources such as staffing, and equipment have a larger effect. The results of this study highlight the need to focus the work environment, rather than individual factors.

### Implications for practice

The results of this study indicate that a key area for intervention is resource adequacy. LPNs reported that having enough staff, equipment, and resources improved their self reported health. This result adds to the evidence supporting suitable nursing staffing level as being essential for nurses’ health, as well as that of patients. Additionally, this result indicates that nurses’ health is impacted by their work environments, not only their own actions. While interventions like exercise and a healthy diet are important, these individual level interventions would fail to address the quality of the work environment. Nurse leaders can be encouraged to secure safe staffing to promote the health of nursing staff at a population level.

### Limitations

Causal inferences cannot be made due to the cross-sectional design of the study. Nevertheless, the findings provide valuable insights into a part of the nursing workforce for which there is limited research. It is acknowledged that other factors like chronic illness have accounted for larger amounts of the variance in other studies. It was beyond the scope of this study to include questions in this area, and it is an acknowledged limitation.

## Conclusion

In conclusion, work environment could play a role in promoting a healthy nursing workforce. With the potential for a global nursing shortage, and negative impacts on nurses during the COVID-19 pandemic, it will be imperative that health care organizations address the health of their workforce. This study identifies areas of the work environment that can have a significant effect on a nurse’s physical and emotional wellbeing. An organization can promote nurse health by ensuring their nursing staff can participate in decision making, have adequate staffing and equipment to perform their work, and encouraging positive team collaborations.

## Supplementary Information

**Additional file 1**: Beta regression models of each sub-scale of the SF-36, and graphs depicting the relationships between SF-36 subscales and demographic variables.

## Data Availability

The data that support the findings of this study are available from the corresponding author upon reasonable request.

## References

[1] Aiken LH, Cimiotti JP, Sloane DM, Smith HL, Flynn L, Neff DF (2011). Effects of nurse staffing and nurse education on patient deaths in hospitals with different nurse work environments. Med Care.

[2] Aiken LH, Clarke SP, Sloane DM, Lake ET, Cheney T (2008). Effects of hospital care environment on patient mortality and nurse outcomes. J Nurs Adm.

[3] Aiken LH, Sloane DM, Clarke S, Poghosyan L, Cho E, You L, Finlayson M, Kanai-Pak M, Aungsuroch Y (2011). Importance of work environments on hospital outcomes in nine countries. Int J Qual Health Care.

[4] Aloba O, Olabisi O, Aloba T (2016). The 10-item connor–davidson resilience scale: factorial structure, reliability, validity, and correlates among student nurses in southwestern Nigeria. J Am Psychiatr Nurses Assoc.

[5] Amsrud KE, Lyberg A, Severinsson E. Development of resilience in nursing students: a systematic qualitative review and thematic synthesis. Nurse Educ Practice. 2019;102621. 10.1016/j.nepr.2019.102621 31726329

[6] Arakawa C, Kanoya Y, Sato C (2011). Factors contributing to medical errors and incidents among hospital nurses—nurses’ health, quality of life, and workplace predict medical errors and incidents—. Ind Health.

[7] Baid H (2018). Resilience in critical care nurses—is it desirable?. Nurs Crit Care.

[8] Bao Y, Bertoia ML, Lenart EB, Stampfer MJ, Willett WC, Speizer FE, Chavarro JE (2016). Origin, methods, and evolution of the three nurses’ health studies. Am J Public Health.

[9] Boev C (2012). The relationship between nurses’ perception of work environment and patient satisfaction in adult critical care. J Nurs Scholarsh.

[10] Canadian Institute for Health Information (2019). Nursing in Canada, 2019.

[11] Chang EM, Daly JW, Hancock KM, Bidewell J, Johnson A, Lambert VA, Lambert CE (2006). The relationships among workplace stressors, coping methods, demographic characteristics, and health in Australian nurses. J Prof Nurs.

[12] Colditz GA, Hankinson SE (2005). The nurses' health study: lifestyle and health among women. Nat Rev Cancer.

[13] Connor KM, Davidson JR (2003). Development of a new resilience scale: the connor-davidson resilience scale (cd-risc). Depress Anxiety.

[14] Cribari-Neto FAZA (2010). Beta regression in r. J Stat Softw.

[15] Davis KG, Kotowski SE (2015). Prevalence of musculoskeletal disorders for nurses in hospitals, long-term care facilities, and home health care: a comprehensive review. Hum Factors.

[16] Dawson AP, McLennan SN, Schiller SD, Jull GA, Hodges PW, Stewart S (2007). Interventions to prevent back pain and back injury in nurses: a systematic review. Occup Environ Med.

[17] Delgado C, Upton D, Ranse K, Furness T, Foster K (2017). Nurses' resilience and the emotional labour of nursing work: an integrative review of empirical literature. Int J Nurs Stud.

[18] Esposito EM, Fitzpatrick JJ (2011). Registered nurses' beliefs of the benefits of exercise, their exercise behaviour and their patient teaching regarding exercise. Int J Nurs Pract.

[19] Faber A, Giver H, Stroyer J, Hannerz H (2010). Are low back pain and low physical capacity risk indicators for dropout among recently qualified eldercare workers? A follow-up study. Scand J Public Health.

[20] Gaskins AJ, Rich-Edwards JW, Lawson CC, Schernhammer ES, Missmer SA, Chavarro JE (2015). Work schedule and physical factors in relation to fecundity in nurses. Occup Environ Med.

[21] Giver H, Faber A, Hannerz H, Stroyer J, Rugulies R (2010). Psychological well-being as a predictor of dropout among recently qualified Danish eldercare workers. Scand J Public Health.

[22] Heidke P, Madsen WL, Langham EM. Registered nurses as role models for healthy lifestyles. AJAN. 2020;37

[23] Hopman WM, Towheed T, Anastassiades T, Tenenhouse A, Poliquin S, Berger C, Joseph L, Brown JP, Murray TM, Adachi JD (2000). Canadian normative data for the sf-36 health survey. CMAJ.

[24] Itzhaki M, Peles-Bortz A, Kostistky H, Barnoy D, Filshtinsky V, Bluvstein I (2015). Exposure of mental health nurses to violence associated with job stress, life satisfaction, staff resilience, and post-traumatic growth. Int J Ment Health Nurs.

[25] Jackson J, Vandall-Walker V, Vanderspank-Wright B, Wishart P, Moore SL (2018). Burnout and resilience in critical care nurses: a grounded theory of managing exposure. Intensive Crit Care Nurs.

[26] Ki J, Ryu J, Baek J, Huh I, Choi-Kwon S (2020). Association between health problems and turnover intention in shift work nurses: health problem clustering. Int J Environ Res Public Health.

[27] Lake ET (2002). Development of the practice environment scale of the nursing work index. Res Nurs Health.

[28] Lauridsen LS, Willert MV, Eskildsen A, Christiansen DH (2017). Cross-cultural adaptation and validation of the danish 10-item connor-davidson resilience scale among hospital staff. Scand J Public Health.

[29] Lawson C, Johnson C, Chavarro J, Lividoti Hibert E, Whelan E, Rocheleau C, Grajewski B, Schernhammer E, Rich-Edwards J (2014). Shift work, long working hours, and physical labour in relation to menstrual function: the nurses' health study 3. Occupat Environ Med.

[30] Lee KJ, Forbes ML, Lukasiewicz GJ, Williams T, Sheets A, Fischer K, Niedner MF (2015). Promoting staff resilience in the pediatric intensive care unit. Am J Crit Care.

[31] Li Y, Cao F, Cao D, Liu J (2015). Nursing students' post-traumatic growth, emotional intelligence and psychological resilience. J Psychiatr Ment Health Nurs.

[32] McDonald G, Jackson D, Vickers MH, Wilkes L (2016). Surviving workplace adversity: a qualitative study of nurses and midwives and their strategies to increase personal resilience. J Nurs Manag.

[33] McGowan JE, Murray K (2016). Exploring resilience in nursing and midwifery students: a literature review. J Adv Nurs.

[34] Michael YL, Colditz GA, Coakley E, Kawachi I (1999). Health behaviors, social networks, and healthy aging: cross-sectional evidence from the nurses' health study. Qual Life Res.

[35] Nyssen A-S, Betastegui P, Braithwaite J, Wears R, Hollnagel E (2017). Is system resilience maintained at the expense of individual resilience?. Resilient health care: reconciling work-as-imagined and work-as-done.

[36] Omrani Z, Talebi E (2018). Quality of life of nurses and related factors. Int J Epidemiol Res.

[37] Parker D, Tuckett A, Eley R, Hegney D (2010). Construct validity and reliability of the practice environment scale of the nursing work index for queensland nurses. Int J Nurs Pract.

[38] Pheasant S, Stubbs D (1992). Back pain in nurses: epidemiology and risk assessment. Appl Ergon.

[39] Priano SM, Hong OS, Chen J-L (2018). Lifestyles and health-related outcomes of us hospital nurses: a systematic review. Nurs Outlook.

[40] Reyes AT, Andrusyszyn MA, Iwasiw C, Forchuk C, Babenko-Mould Y (2015). Nursing students' understanding and enactment of resilience: a grounded theory study. J Adv Nurs.

[41] Rushton CH, Batcheller J, Schroeder K, Donohue P (2015). Burnout and resilience among nurses practicing in high-intensity settings. Am J Crit Care.

[42] Schluter P, Turner C, Huntington A, Bain C, Mcclure RJ (2011). Work/life balance and health: the nurses and midwives e-cohort study. Int Nurs Rev.

[43] Stasinopoulos M. Generalised additive models for location scale and shape; 2016

[44] Taylor RA (2019). Contemporary issues: resilience training alone is an incomplete intervention. Nurse Educ Today.

[45] Trudel-Fitzgerald C, Chen Y, Singh A, Okereke OI, Kubzansky LD (2016). Psychiatric, psychological, and social determinants of health in the nurses’ health study cohorts. Am J Public Health.

[46] Turner SB (2015). Resilience of nurses in the face of disaster. Disaster Med Public Health Prep.

[47] Waehrer G, Leigh JP, Miller TR (2005). Costs of occupational injury and illness within the health services sector. Int J Health Serv.

[48] Ware J, Kosinski M, Keller S. Sf-36 physical and mental health summary scales. A user's manual**,** 1994; 2001.

[49] World Health Organization 2019. Gender equity in the health workforce: Analysis of 104 countries. World Health Organization.

