# Supplementary material for: The impact of the work environment on the health-related quality of life of Licensed Practical Nurses: a cross-sectional survey in four work environments
Source: Health Qual Life Outcomes. 2022 Mar 19;20:44. doi: 10.1186/s12955-022-01951-9 (PMC8934126; doi:10.1186/s12955-022-01951-9)

**Table 1a:** Predictors of body pain using beta regression model.

| Covariates | Mean sub-model (logit link) | | | Precision sub model (log link) | | |
| --- | --- | --- | --- | --- | --- | --- |
|  | Odds ratio | Conf. Interval | P-value | Estimate | Standard error | P-value |
| Intercept | 0.75 | 0.53,1.17 | 0.110 | 0.635 | 0.182 | **0.000** |
| Leadership | 1.04 | 0.96,1.12 | 0.326 | -0.015 | 0.039 | 0.704 |
| Resource | 1.17 | 1.10,1.24 | **0.000** | -0.024 | 0.035 | 0.495 |
| Relationship | 1.06 | 0.98,1.15 | 0.126 | 0.070 | 0.042 | 0.093 |
| Resilience | 1.03 | 1.02,1.04 | **0.000** | 0.002 | 0.004 | 0.708 |
| Male (ref: female) | 1.20 | 0.98,1.46 | 0.066 | -0.108 | 0.101 | 0.286 |
| Area of work (ref: Acute care) |  |  |  |  |  |  |
| Continuing care | 1.03 | 0.91,1.16 | 0.589 | -0.009 | 0.062 | 0.884 |
| Community care | 1.21 | 1.05,1.39 | **0.006** | 0.063 | 0.072 | 0.384 |
| Primary care | 1.06 | 0.92,1.21 | 0.440 | -0.085 | 0.072 | 0.239 |
| Year of practice (ref: 0 -5 years) |  |  |  |  |  |  |
| 6 – 15 years | 1.13 | 1.01,1.26 | **0.029** | 0.052 | 0.059 | 0.377 |
| 16 plus years | 0.89 | 0.75,1.05 | 0.177 | -0.006 | 0.086 | 0.941 |
| Employment status (ref: fulltime) |  |  |  |  |  |  |
| Part time | 1.09 | 0.99,1.21 | 0.089 | -0.001 | 0.053 | 0.988 |
| Casual | 1.27 | 1.11,1.46 | **0.000** | 0.006 | 0.072 | 0.933 |
| Participant age | 0.99 | 0.98,0.99 | **0.003** | -0.005 | 0.003 | 0.069 |

**Table 1b:** Predictors of emotional well-being using beta regression model.

| Covariates | Mean sub-model (logit link) | | | Precision sub model (log link) | | |
| --- | --- | --- | --- | --- | --- | --- |
|  | Odds ratio | Conf. Interval | P-value | Estimate | Standard error | P-value |
| Intercept | 0.10 | 0.08, 0.13 | **0.000** | 2.948 | 0.185 | **0.000** |
| Leadership | 1.05 | 1.00, 1.10 | 0.050 | -0.082 | 0.040 | **0.041** |
| Resource | 1.11 | 1.07, 1.16 | **0.000** | -0.010 | 0.034 | 0.765 |
| Relationship | 1.07 | 1.02, 1.13 | **0.004** | 0.084 | 0.042 | 0.053 |
| Resilience | 1.08 | 1.07, 1.08 | **0.000** | -0.024 | 0.004 | **0.000** |
| Male (ref: female) | 1.09 | 0.97, 1.23 | 0.152 | -0.003 | 0.096 | 0.971 |
| Area of work (ref: Acute care) |  |  |  |  |  |  |
| Continuing care | 1.01 | 0.94, 1.09 | 0.843 | -0.105 | 0.062 | 0.089 |
| Community care | 0.89 | 0.82, 0.96 | **0.003** | 0.003 | 0.070 | 0.967 |
| Primary care | 0.94 | 0.87, 1.03 | 0.181 | -0.147 | 0.071 | **0.039** |
| Year of practice  (ref: 0 - 5years) |  |  |  |  |  |  |
| 6 – 15 years | 0.97 | 0.91, 1.04 | 0.375 | 0.114 | 0.058 | **0.047** |
| 16 plus years | 0.91 | 0.82, 1.01 | 0.090 | 0.342 | 0.087 | **0.000** |
| Employment status (ref:fulltime) |  |  |  |  |  |  |
| Part time | 1.04 | 0.98, 1.11 | 0.231 | 0.080 | 0.053 | 0.127 |
| Casual | 1.15 | 1.06, 1.25 | **0.001** | 0.161 | 0.069 | **0.020** |
| Participant age | 1.01 | 1.01, 1.02 | **0.000** | -0.009 | 0.003 | **0.001** |

**Table 1c:** Predictors of general health using beta regression model

| Covariates | Mean sub-model (logit link) | | | Precision sub model (log link) | | |
| --- | --- | --- | --- | --- | --- | --- |
|  | Odds ratio | Conf. Interval | P-value | Estimate | Standard error | P-value |
| Intercept | 0.14 | 0.11,0.19 | **0.000** | 2.1407 | 0.1797 | **0.000** |
| Leadership | 1.05 | 0.99,1.10 | 0.125 | 0.1101 | 0.0390 | **0.004** |
| Resource | 1.08 | 1.03,1.13 | **0.002** | 0.0404 | 0.0336 | 0.228 |
| Relationship | 1.10 | 1.04,1.16 | **0.001** | -0.1453 | 0.0413 | **0.000** |
| Resilience | 1.06 | 1.06,1.07 | **0.000** | -0.0253 | 0.0044 | **0.000** |
| Male (ref: female) |  |  |  | -0.1772 | 0.0937 | 0.058 |
| Area of work (ref: Acute care) | 1.09 | 0.94,1.24 | 0.275 |  |  |  |
| Continuing care | 1.14 | 1.05,1.23 | **0.004** | 0.0275 | 0.0606 | 0.649 |
| Community care | 0.93 | 0.83,1.03 | 0.133 | 0.1127 | 0.0682 | 0.098 |
| Primary care | 0.98 | 0.88,1.08 | 0.711 | 0.0401 | 0.0693 | 0.562 |
| Year of practice (ref: 0 - 5years) |  |  |  |  |  |  |
| 6 – 15 years | 0.94 | 0.86,1.03 | 0.167 | 0.0454 | 0.0563 | 0.419 |
| 16 plus years | 0.84 | 0.71,0.97 | **0.006** | -0.0329 | 0.0850 | 0.699 |
| Employment status (ref: fulltime) |  |  |  |  |  |  |
| Part time | 1.08 | 1.01,1.16 | **0.043** | 0.0877 | 0.0514 | 0.087 |
| Casual | 1.17 | 1.07,1.27 | **0.002** | -0.0523 | 0.0677 | 0.440 |
| Participant age | 1.01 | 1.01,1.01 | **0.000** | -0.0002 | 0.0027 | 0.927 |

**Table 1d:** Predictors of energy fatigue using a beta regression model

| Covariates | Mean sub-model (logit link) | | | Precision sub model (log link) | | |
| --- | --- | --- | --- | --- | --- | --- |
|  | Odds ratio | Conf. Interval | P-value | Estimate | Standard error | P-value |
| Intercept | 0.05 | 0.04,0.06 | **0.000** | 1.653 | 0.178 | **0.000** |
| Leadership | 1.12 | 1.07,1.17 | **0.000** | 0.136 | 0.039 | **0.000** |
| Resource | 1.25 | 1.20,1.30 | **0.000** | 0.001 | 0.033 | 0.981 |
| Relationship | 0.99 | 0.94,1.04 | 0.791 | 0.052 | 0.041 | 0.204 |
| Resilience | 1.06 | 1.05,1.07 | **0.000** | -0.019 | 0.004 | **0.000** |
| Male (ref: female) | 1.24 | 1.12,1.38 | **0.000** | 0.085 | 0.092 | 0.358 |
| Area of work (ref: Acute care) |  |  |  |  |  |  |
| Continuing care | 1.06 | 0.99,1.14 | 0.097 | -0.038 | 0.059 | 0.522 |
| Community care | 1.02 | 0.94,1.10 | 0.601 | 0.113 | 0.068 | 0.095 |
| Primary care | 1.02 | 0.94,1.10 | 0.683 | 0.097 | 0.069 | 0.158 |
| Year of practice (ref: 0 - 5years) |  |  |  |  |  |  |
| 6 – 15 years | 0.86 | 0.80,0.92 | **0.000** | 0.015 | 0.056 | 0.795 |
| 16 plus years | 0.81 | 0.73,0.90 | **0.000** | -0.191 | 0.084 | **0.022** |
| Employment status (ref: fulltime) |  |  |  |  |  |  |
| Part time | 1.11 | 1.05,1.18 | **0.001** | 0.129 | 0.051 | **0.011** |
| Casual | 1.25 | 1.16,1.35 | **0.000** | 0.299 | 0.067 | **0.000** |
| Participant age | 1.02 | 1.01,1.02 | **0.000** | 0.000 | 0.003 | 0.940 |

**Table 1e:** Predictors of role physical using one inflated beta regression model.

|  | Mean (logit link)** | | Sigma (log link) | | NU (logit link)* | |
| --- | --- | --- | --- | --- | --- | --- |
| Covariates | OR, 95% CI | P-value | Estimate (SE) | P value | OR, 95% CI | P-value |
| Intercept | 0.19 (0.10,0.36) | **0.000** | -0.156 (0.275) | 0.569 | -3.028 (0.307) | **0.000** |
| Leadership | 1.05 (0.92,1.19) | 0.480 | 0.062 (0.059) | 0.294 | 0.079 (0.065) | 0.224 |
| Resource | 1.11 (0.99,1.24) | 0.058 | 0.058 (0.052) | 0.265 | 0.278 (0.056) | **0.000** |
| Relationship | 1.07 (0.93,1.23) | 0.330 | -0.023 (0.064) | 0.722 | 0.111 (0.068) | 0.101 |
| Resilience | 1.03 (1.01,1.04) | **0.001** | 0.007 (0.007) | 0.285 | 0.071 (0.007) | **0.000** |
| Male (ref: female) | 0.99 (0.72,1.37) | 0.972 | 0.214 (0.157) | 0.172 | 0.138 (0.160) | 0.388 |
| Area of work  (ref: Acute care) |  |  |  |  |  |  |
| Continuing care | 1.07 (0.87,1.32) | 0.507 | 0.062 (0.094) | 0.508 | 0.028 (0.101) | 0.779 |
| Community care | 0.92 (0.72,1.17) | 0.478 | -0.069 (0.109) | 0.528 | 0.011 (0.115) | 0.921 |
| Primary care | 0.94 (0.74,1.19) | 0.644 | 0.099 (0.113) | 0.383 | 0.113 (0.118) | 0.340 |
| Year of practice  (ref: 0 - 5years) |  |  |  |  |  |  |
| 6 – 15 years | 0.97 (0.80,1.18) | 0.764 | -0.027 (0.091) | 0.766 | 0.007 (0.095) | 0.944 |
| 16 plus years | 1.03 (0.77,1.39) | 0.832 | 0.008 (0.137) | 0.955 | -0.074 (0.143) | 0.603 |
| Employment status  (ref: fulltime) |  |  |  |  |  |  |
| Part time | 1.00 (0.84,1.20) | 0.960 | 0.032 (0.080) | 0.689 | 0.141 (0.086) | 0.099 |
| Casual | 0.96 (0.75,1.23) | 0.735 | 0.012 (0.113) | 0.916 | 0.415 (0.116) | **0.000** |
| Participant age | 0.99 (0.98,0.99) | **0.039** | -0.007 (0.004) | 0.095 | 0.002 (0.004) | 0.599 |

*Nu (logit link) indicates predictors of the optimal health in log-odds values,

** Indicates predictors of suboptimal health using OR; *SE,* Standard errors; *OR,* Odds ratios; *CI,* Confidence interval; *Bolded,* Indicate statistical significance (at the 5 % level); Ref, Reference category.

**Table 1d:** Predictors of social functioning using a one inflated beta regression model.

|  | Mean (logit link)** | | Sigma (log link) | | NU (logit link)* | |
| --- | --- | --- | --- | --- | --- | --- |
| Covariates | OR, 95% CI | P-value | Estimate (SE) | P value | Estimate (SE) | P-value |
| Intercept | 0.33 (0.25,0.44) | **0.000** | 0.373 (0.202) | 0.064 | -6.104 (0.347) | **0.000** |
| Leadership | 1.03 (0.97,1.09) | 0.353 | 0.045 (0.047) | 0.341 | -0.038 (0.068) | 0.573 |
| Resource | 1.14 (1.09,1.19) | **0.000** | 0.172 (0.040) | **0.000** | 0.263 (0.057) | **0.000** |
| Relationship | 1.04 (0.97,1.10) | 0.293 | -0.018 (0.046) | 0.701 | 0.271 (0.074) | **0.000** |
| Resilience | 1.03 (1.02,1.04) | **0.000** | 0.023 (0.006) | **0.000** | 0.098 (0.008) | **0.000** |
| Male (ref: female) | 1.12 (0.98,1.29) | 0.116 | 0.201 (0.120) | 0.095 | 0.076 (0.155) | 0.625 |
| Area of work  (ref: Acute care) |  |  |  |  |  |  |
| Continuing care | 1.02 (0.93,1.12) | 0.691 | 0.011 (0.076) | 0.880 | -0.056 (0.103) | 0.589 |
| Community care | 0.91 (0.79,1.02) | 0.088 | -0.386 (0.086) | **0.000** | -0.210 (0.117) | 0.073 |
| Primary care | 0.90 (0.80,1.01) | 0.070 | -0.371 (0.090) | **0.000** | -0.114 (0.119) | 0.339 |
| Year of practice  (ref: 0 - 5years) |  |  |  |  |  |  |
| 6 – 15 years | 1.15 (1.05,1.25) | **0.003** | 0.173 (0.075) | **0.021** | 0.028 (0.097) | 0.771 |
| 16 plus years | 1.00 (0.86,1.17) | 0.967 | -0.217 (0.118) | 0.065 | 0.255 (0.141) | 0.071 |
| Employment status  (ref: fulltime) |  |  |  |  |  |  |
| Part time | 1.00 (0.92,1.08) | 0.940 | -0.185 (0.063) | **0.003** | 0.166 (0.088) | 0.060 |
| Casual | 1.20 (1.08,1.33) | **0.001** | 0.209 (0.086) | **0.016** | 0.354 (0.115) | **0.002** |
| Participant age | 1.00 (1.00,1.01) | 0.133 | 0.002 (0.004) | 0.574 | 0.020 (0.004) | **0.000** |

*Nu (logit link) indicates predictors of the optimal health in log-odds values,

** Indicates predictors of suboptimal health using OR; *SE,* Standard errors; *OR,* Odds ratios; *CI,* Confidence interval; *Bolded,* Indicate statistical significance (at the 5 % level); Ref, Reference category.

**Table 1f:** Predictors of physical functioning using one inflated beta regression model

|  | Mean (logit link)** | | Sigma (log link) | | NU (logit link)* | |
| --- | --- | --- | --- | --- | --- | --- |
| Covariates | OR, 95% CI | P-value | Estimate (SE) | P value | Estimate (SE) | P-value |
| Intercept | 2.43 (1.71,3.46) | **0.000** | 1.427 (0.227) | **0.000** | -1.400 (0.301) | **0.000** |
| Leadership | 0.97 (0.89,1.05) | 0.436 | -0.061 (0.055) | 0.262 | 0.031 (0.065) | 0.627 |
| Resource | 1.02 (0.95,1.09) | 0.650 | -0.018 (0.046) | 0.691 | 0.139 (0.055) | **0.011** |
| Relationship | 1.16 (1.08,1.25) | **0.001** | 0.228 (0.059) | **0.000** | 0.020 (0.069) | 0.777 |
| Resilience | 1.00 (0.99,1.01) | 0.887 | -0.034 (0.005) | **0.000** | 0.065 (0.007) | **0.000** |
| Male (ref: female) | 0.95 (0.76,1.17) | 0.623 | -0.17 (0.130) | 0.192 | 0.301 (0.147) | **0.040** |
| Area of work  (ref: Acute care) |  |  |  |  |  |  |
| Continuing care | 0.91 (0.78,1.04) | 0.144 | -0.104 (0.086) | 0.223 | -0.102 (0.099) | 0.301 |
| Community care | 1.00 (0.87,1.15) | 0.958 | 0.138 (0.096) | 0.150 | -0.161 (0.112) | 0.150 |
| Primary care | 0.97 (0.84,1.12) | 0.700 | 0.015 (0.094) | 0.875 | -0.022 (0.114) | 0.846 |
| Year of practice  (ref: 0 - 5years) |  |  |  |  |  |  |
| 6 – 15 years | 1.04 (0.92,1.17) | 0.555 | 0.029 (0.082) | 0.724 | 0.022 (0.091) | 0.808 |
| 16 plus years | 0.91 (0.76,1.09) | 0.312 | -0.095 (0.126) | 0.452 | -0.072 (0.143) | 0.614 |
| Employment status  (ref: fulltime) |  |  |  |  |  |  |
| Part time | 1.11 (1.01,1.23) | **0.048** | 0.161 (0.068) | **0.017** | -0.069 (0.085) | 0.413 |
| Casual | 1.20 (1.04,1.37) | **0.013** | 0.247 (0.092) | **0.008** | 0.061 (0.110) | 0.577 |
| Participant age | 1.00 (0.99,1.00) | 0.074 | 0.007 (0.004) | **0.058** | -0.040 (0.005) | **0.000** |

*Nu (logit link) indicates predictors of the optimal health in log-odds values,

** Indicates predictors of suboptimal health using OR; *SE,* Standard errors; *OR,* Odds ratios; *CI,* Confidence interval; *Bolded,* Indicate statistical significance (at the 5 % level); Ref, Reference category.

**Table 1g:** Predictors of role emotional using one inflated beta regression model.

|  | Mean (logit link)** | | Sigma (log link) | | NU (logit link)* | |
| --- | --- | --- | --- | --- | --- | --- |
| Covariates | OR, 95% CI | P-value | Estimate (SE) | P value | Estimate (SE) | P-value |
| Intercept | 0.11 (0.06,0.20) | 0.000 | -0.224 (0.296) | 0.449 | -5.104 (0.336) | **0.000** |
| Leadership | 0.94 (0.82,1.08) | 0.367 | -0.077 (0.063) | 0.220 | 0.100 (0.067) | 0.137 |
| Resource | 1.10 (0.98,1.24) | 0.108 | 0.038 (0.054) | 0.480 | 0.450 (0.059) | **0.000** |
| Relationship | 1.13 (0.98,1.30) | 0.088 | 0.081 (0.067) | 0.225 | -0.068 (0.071) | 0.337 |
| Resilience | 1.03 (1.02,1.05) | **0.000** | 0.011 (0.007) | 0.143 | 0.112 (0.008) | **0.000** |
| Male (ref: female) | 0.73 (0.49,1.08) | 0.121 | -0.181 (0.183) | 0.322 | 0.143 (0.169) | 0.399 |
| Area of work  (ref: Acute care) |  |  |  |  |  |  |
| Continuing care | 0.88 (0.70,1.10) | 0.258 | -0.066 (0.104) | 0.528 | 0.123 (0.106) | 0.245 |
| Community care | 0.89 (0.70,1.14) | 0.371 | -0.040 (0.115) | 0.727 | -0.067 (0.119) | 0.573 |
| Primary care | 0.77 (0.59,0.98) | **0.040** | -0.060 (0.120) | 0.614 | -0.068 (0.121) | 0.577 |
| Year of practice  (ref: 0 - 5years) |  |  |  |  |  |  |
| 6 – 15 years | 1.05 (0.85,1.28) | 0.632 | 0.012 (0.095) | 0.901 | -0.069 (0.098) | 0.479 |
| 16 plus years | 1.11 (0.81,1.52) | 0.508 | 0.026 (0.146) | 0.859 | -0.269 (0.151) | 0.075 |
| Employment status  (ref: fulltime) |  |  |  |  |  |  |
| Part time | 0.99 (0.82,1.19) | 0.959 | -0.003 (0.087) | 0.972 | 0.228 (0.090) | **0.011** |
| Casual | 1.02 (0.79,1.29) | 0.894 | 0.019 (0.114) | 0.865 | 0.267 (0.118) | **0.024** |
| Participant age | 1.00 (0.99,1.01) | 0.988 | -0.001 (0.005) | 0.772 | 0.029 (0.005) | **0.000** |

*Nu (logit link) indicates predictors of the optimal health in log-odds values,

** Indicates predictors of suboptimal health using OR; *SE,* Standard errors; *OR,* Odds ratios; *CI,* Confidence interval; *Bolded,* Indicate statistical significance (at the 5 % level); Ref, Reference category.


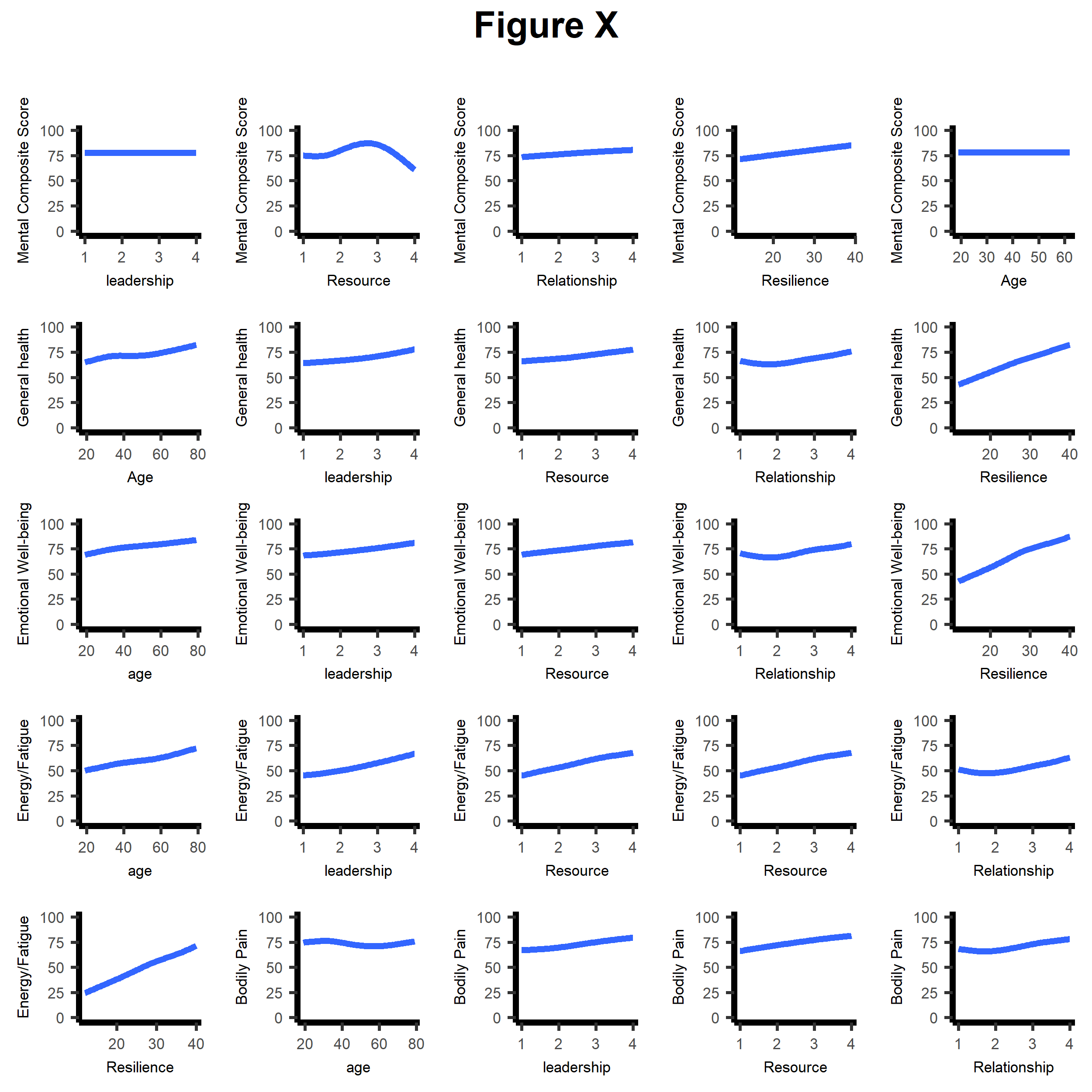


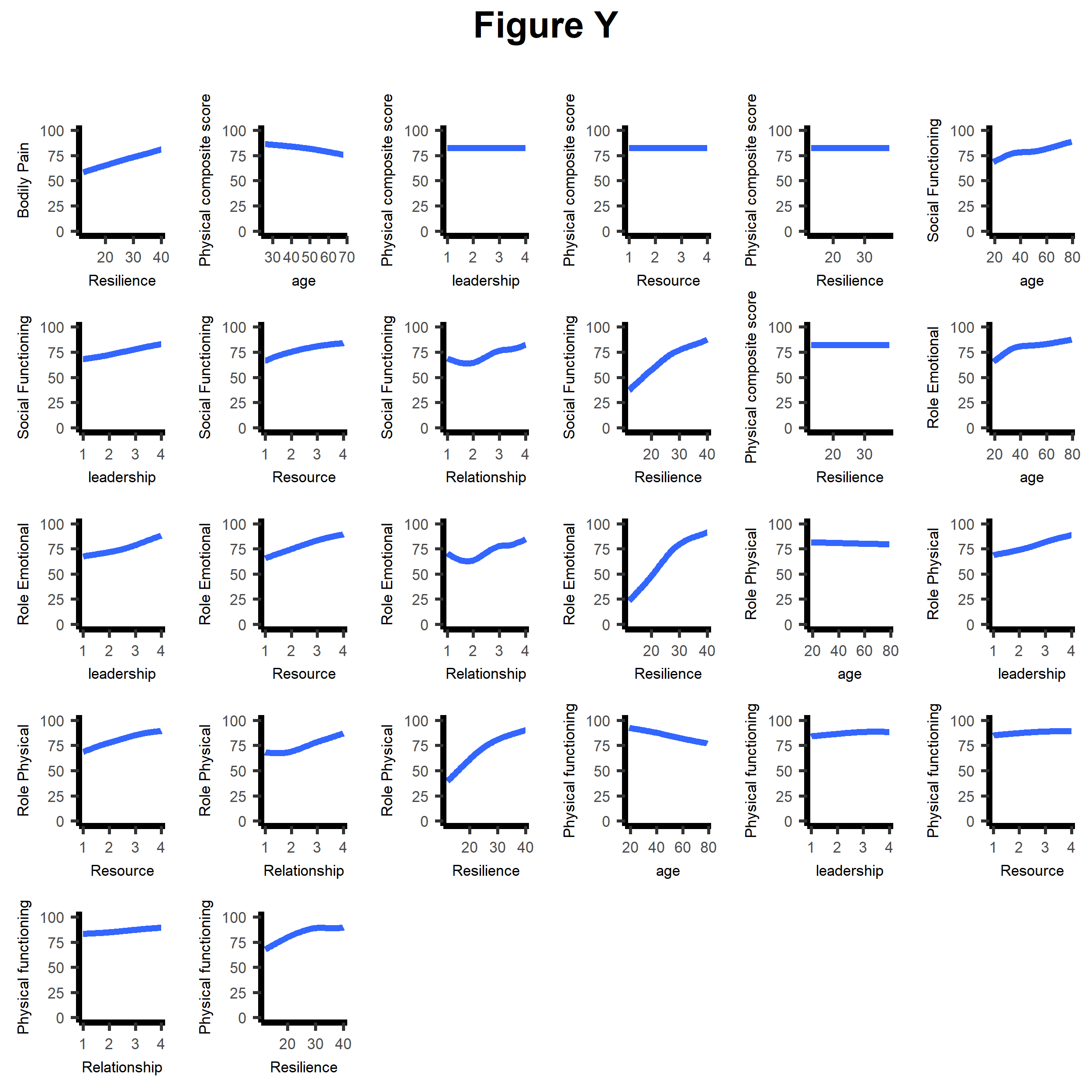

Supplement: Supplementary file 1 — Additional file 1: Beta regression models of each sub-scale of the SF-36, and graphs depicting the relationships between SF-36 subscales and demographic variables. [file 12955_2022_1951_MOESM1_ESM.docx]
